# Inpatient Gastroenterology Workup Prior to Transesophageal Echocardiogram Is of Minimal Benefit to Patients

**DOI:** 10.1016/j.gastha.2023.03.011

**Published:** 2023-03-14

**Authors:** J. Mizrahi, L. Klaychman, A. Paradkar, E. Menashe, J. Marhaba, D. Jamorabo

**Affiliations:** 1 Division of Gastroenterology and Hepatology, Stony Brook University Hospital, Stony Brook, New York; 2 Department of Medicine, Stony Brook University Hospital, Stony Brook, New York

See editorial on page 743.

Despite the low incidence of complications from transesophageal echocardiograms (TEEs),^1^ gastroenterologists may be consulted to evaluate patients prior to TEE when patients have gastrointestinal (GI) comorbidities. There are broad, relative GI-related contraindications for TEEs, including a history of dysphagia, recent upper GI bleeding, and esophageal varices.^2^ Most studies that have investigated the utility of the GI service’s involvement have focused on patients with cirrhosis and found that TEEs could be performed safely in such individuals.^3^, ^4^, ^5^ Other studies have examined the utility of performing an esophagogastroduodenoscopy (EGD) prior to TEEs and arrived at mixed results.^6^, ^7^, ^8^ To our knowledge, there are no studies assessing the utility of a GI workup prior to a TEE, regardless of the GI team’s interventions.

This was a retrospective cohort study of hospitalized patients from January 01, 2012, to December 31, 2020 at our institution for whom the cardiology team requested the GI team’s clearance prior to doing a TEE. We collected data on patient demographics, indications for both the TEE and the GI consult, GI team recommendations, and complications from the TEE. Our team measured length of stay as well as carried out a subgroup analysis comparing patients whom the GI service cleared for the procedure without any further workup vs patients for whom the GI consultant recommended a workup.

We identified 79 inpatients, of which 51 were men (64.6%) and 70 (88.6%) were White. The average age of our cohort was 65.6 years ( ± 13.2 years), and the average length of stay was 2.97 days. We found that 44.3% of patients had no prior GI diagnosis and 56.96% had no GI symptoms at the time of the consult. Most GI consult requests were for dysphagia/odynophagia (29.1%) or a history of GI bleeding/anemia (27.8%) (Figure). 24.1% of patients had had an EGD within a year of the TEE, and 12.7% of patients had prior radiographic or endoscopic evidence of esophageal varices.

**Figure:**
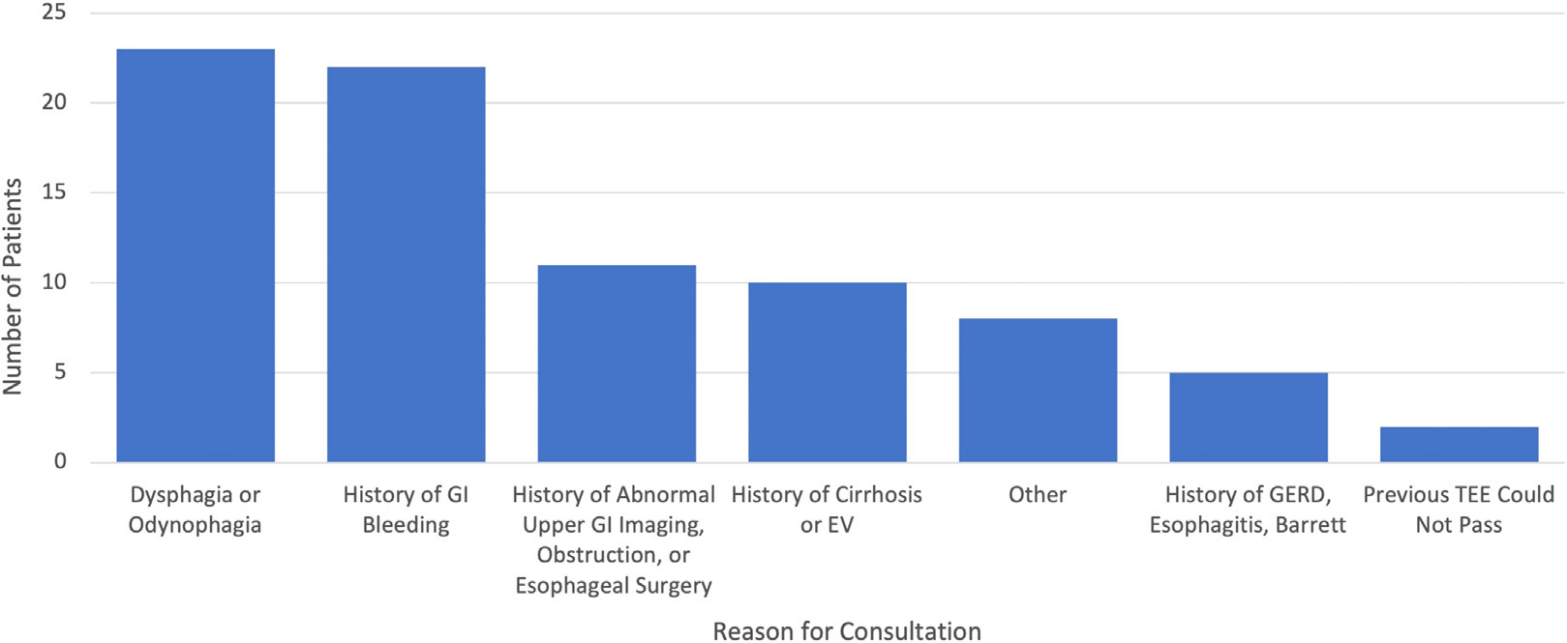
Indications for GI consult prior to TEE. GI, gastrointestinal; TEE, transesophageal echocardiogram; EV, esophageal varices; GERD, gastroesophageal reflux disease.

The GI team initiated an inpatient workup in 57 (72.2%) patients, which included 49 (85.9%) EGDs and 9 (15.8%) esophagrams or computed tomographies. 45/79 (56.9%) patients had no active GI symptoms, yet 23/45 of those patients (51.1%) still underwent an EGD and 4/45 patients (8.8%) had GI imaging. Of the 34/79 (43.1%) patients who had active GI symptoms at the time of GI consultation, 26/34 (76.5%) had an EGD and 5/34 (14.7%) had imaging done.

All the imaging was normal except for one esophagram, which showed a tortuous esophagus. Of the 49 EGDs performed, 19 (38.8%) were normal with 7 (14.3%) revealing esophagitis, 5 (10.2%) showing small varices, and 3 (6.1%) visualizing hiatal hernias. All but one of the EGDs (48/49; 97.9%) resulted in clearance of the patient for TEE. One patient had a Forest Class 1B duodenal ulcer, which was treated appropriately, and the TEE was performed 5 days later with no complications.

There were zero readmissions within 30 days related to the TEE or to a GI indication. There were zero EGD or TEE complications in our cohort.

We performed a subgroup analysis comparing patients whom the GI team cleared for TEE without further workup to those whom the GI service advised further workup (Table). Patients in whom an EGD was performed within the year preceding the TEE were 6.12 times more likely to be cleared by GI without any further workup (95% confidence interval 2.04–19.6; *P*-value < .01), while those with active GI symptoms had a 72% less chance of getting cleared without any further workup (odds ratio 0.28; 95% CI 0.08–0.83; *P*-value .03). No other variables were significantly associated with the GI team clearing patients for TEE with or without a further workup. The mean time from GI consult to TEE being done was higher in patients who required a workup prior to clearance compared to patients whom GI cleared (3.35 days vs 2 days).

**Table:** Multivariate Analysis Comparing Patients Whom the GI Team Cleared for a TEE Without Further Workup to Those for Whom the GI Service Advised Further Studies

Variable	Did GI clear patient for TEE without further tests?		OR (95% CI)	*P*-value
	Yes (n = 22)	No (n = 57)		
Sex				
Female	8	20	Ref	.92
Male	14	37	0.946 (0.34–2.72)	
Race				
Asian	0	1	None	N/A
Black	0	3		
Hispanic	0	5		
White	22	48		
Preexisting GI diagnosis				
Yes	13	31	1.21 (0.45–3.36)	.71
No	9	26	Ref	
Prior upper GI surgery				
Yes	4	7	1.59 (0.38–5.92)	.5
No	18	50	Ref	
EGD within 1 y of GI consult				
Yes	11	8	6.12 (2.04–19.6)	<.01
No	11	49	Ref	
Upper GI imaging within 1 y of GI consult				
Yes	11	29	0.97 (0.36–2.6)	.94
No	11	28	Ref	
Previous evidence of esophageal varices (imaging or EGD)				
Yes	3	7	1.13 (0.23–4.53)	.87
No	19	50	Ref	
GI symptoms prior to GI team evaluation				
Yes	5	29	0.28 (0.08–0.83)	.03
No	17	28	Ref	
Was GI evaluation sought within a month after TEE				
Yes	1	3	0.86 (0.04–7.13)	.9
No	21	54	Ref	
30-d readmission after TEE				
Yes	1	4	0.63 (0.03–4.58)	.69
No	21	53	Ref	

OR, odds ratio; CI, confidence interval.

To validate our findings, we matched the 79 patients who did receive a GI consult prior to TEE in our study to 79 randomly selected patients who did not receive a GI consult prior to TEE from 2021 to 2022 at our institution. While age (65.6 years, standard deviation 13.2 vs 70 years, SD 12.5, *P*-value .03) and some cardiac indications for the TEE were statistically different between the 2 groups, the 2 groups were otherwise largely similar, and the few differences between them did not denote a clinically significant difference (Table A1).

The decision to work up a patient prior to clearing them for TEE is often subjective, and thus we sought to identify predictive variables that may be associated with inpatient GI clearance and further workup. Multivariate analysis showed that the GI team tended to clear patients with prior EGDs within the previous year and to work up patients with active GI symptoms when controlling for all studied variables. This finding is not surprising—active symptoms generally require investigation whereas patients with a recent GI investigation may not. However, our finding that 51.1% of patients without active GI symptoms still underwent an EGD while another 8.8% had GI imaging is notable and could reflect either pressure placed upon the GI team to carry out a workup or undue caution from the GI team itself.

Determining objective criteria for initiating an inpatient workup prior to a TEE is crucial given that diagnostic interventions increase a patient’s hospital bill and drive-up healthcare costs. It is also well established that patients experience significant anxiety and even depression while hospitalized,^9^^,^^10^ and thus it is important to avoid procedures and tests that are not medically appropriate while hospitalized. Our findings underscore the need to formulate a standardized approach to deciding whether or not to work up a patient with GI comorbidities prior to TEE.

We conclude that hospital resources—procedural, radiographic, and logistical—were likely wasted by obtaining a GI team evaluation given the low risk of complications. Further plans include a prospective study of TEEs performed to identify predictive factors for both GI consultation and GI-related TEE complications, with the goal of identifying objective criteria that warrant GI team clearance prior to TEE.

## Supplementary materials

Table A1
