# Supplementary material for: Inpatient Gastroenterology Workup Prior to Transesophageal Echocardiogram Is of Minimal Benefit to Patients
Source: Gastro Hep Adv. 2023 Mar 14;2(5):639–41. doi: 10.1016/j.gastha.2023.03.011 (PMC11307618; doi:10.1016/j.gastha.2023.03.011)
Supplement: Table A1 [file mmc1.docx]

Table A1 - Comparison of Original Retrospective Patients (Inpatient GI Team Consult) vs 2021-2022 Patients (No Inpatient Team GI Consult)

|  | **2021-2022** |  | **Retrospective** |  | **Test % diff** |
| --- | --- | --- | --- | --- | --- |
| **Var** | **N** | **%** | **N** | **%** | **Pval (chi-sq)** |
| **Sex** |  |  |  |  |  |
| Female | 24 | [30.4] | 28 | [35.4] | 0.61 |
| Male | 55 | [69.6] | 51 | [64.6] |  |
| **Race** |  |  |  |  |  |
| Non-White | 9 | [11.4] | 9 | [11.4] | 1 |
| White | 70 | [88.6] | 70 | [88.6] |  |
| **Insurance** |  |  |  |  |  |
| Blue Cross | 8 | [10.1] | 4 | [5.06] | 0.12 |
| Commercial | 7 | [8.86] | 8 | [10.1] |  |
| Commercial/HMO | 3 | [3.8] | 6 | [7.59] |  |
| Medicaid | 4 | [5.06] | 9 | [11.4] |  |
| Medicaid MGD Care | 6 | [7.59] | 3 | [3.8] |  |
| Medicare | 42 | [53.2] | 47 | [59.5] |  |
| Self Pay | 9 | [11.4] | 2 | [2.53] |  |
| **Indication.for.TEE..Neuro.R.O.cardiac.source.of.embolus.** |  |  |  |  |  |
| NO | 75 | [94.9] | 73 | [92.4] | 0.74 |
| YES | 4 | [5.06] | 6 | [7.59] |  |
| **Indication.for.TEE..Other.Cardio.** |  |  |  |  |  |
| NO | 4 | [5.06] | 6 | [7.59] | 0.74 |
| YES | 75 | [94.9] | 73 | [92.4] |  |
| **Preexisting.Cardiology.Comorbidities..choice.CAD.** |  |  |  |  |  |
| NO | 43 | [54.4] | 45 | [57] | 0.87 |
| YES | 36 | [45.6] | 34 | [43] |  |
| **Preexisting.Cardiology.Comorbidities..choice.HFrEF.** |  |  |  |  |  |
| NO | 66 | [83.5] | 62 | [78.5] | 0.54 |
| YES | 13 | [16.5] | 17 | [21.5] |  |
| **Preexisting.Cardiology.Comorbidities..choice.HFpEF.** |  |  |  |  |  |
| NO | 69 | [87.3] | 75 | [94.9] | 0.16 |
| YES | 10 | [12.7] | 4 | [5.06] |  |
| **Preexisting.Cardiology.Comorbidities..choice.Atrial.Fibrillation.** |  |  |  |  |  |
| NO | 34 | [43] | 48 | [60.8] | 0.038 |
| YES | 45 | [57] | 31 | [39.2] |  |
| **Preexisting.Cardiology.Comorbidities..choice.Atrial.Flutter.** |  |  |  |  |  |
| NO | 70 | [88.6] | 74 | [93.7] | 0.4 |
| YES | 9 | [11.4] | 5 | [6.33] |  |
| **Preexisting.Cardiology.Comorbidities..choice.Other.Arrhythmia.** |  |  |  |  |  |
| NO | 70 | [88.6] | 70 | [88.6] | 1 |
| YES | 9 | [11.4] | 9 | [11.4] |  |
| **Preexisting.Cardiology.Comorbidities..choice.Moderate.Severe.AS.** |  |  |  |  |  |
| NO | 72 | [91.1] | 71 | [89.9] | 1 |
| YES | 7 | [8.86] | 8 | [10.1] |  |
| **Preexisting.Cardiology.Comorbidities..choice.Moderate.Severe.AR.** |  |  |  |  |  |
| NO | 78 | [98.7] | 77 | [97.5] | 1 |
| YES | 1 | [1.27] | 2 | [2.53] |  |
| **Preexisting.Cardiology.Comorbidities..choice.Moderate.Severe.MR.** |  |  |  |  |  |
| NO | 69 | [87.3] | 66 | [83.5] | 0.65 |
| YES | 10 | [12.7] | 13 | [16.5] |  |
| **Preexisting.Cardiology.Comorbidities..choice.Moderate.Severe.MS.** |  |  |  |  |  |
| NO | 79 | [100] | 78 | [98.7] | 1 |
| YES | 0 | [0] | 1 | [1.27] |  |
| **Preexisting.Cardiology.Comorbidities..choice.Moderate.Severe.TR.** |  |  |  |  |  |
| NO | 78 | [98.7] | 77 | [97.5] | 1 |
| YES | 1 | [1.27] | 2 | [2.53] |  |
| **Preexisting.Cardiology.Comorbidities..choice.Pulmonary.HTN.** |  |  |  |  |  |
| NO | 76 | [96.2] | 75 | [94.9] | 1 |
| YES | 3 | [3.8] | 4 | [5.06] |  |
| **Preexisting.Cardiology.Comorbidities..choice.RV.Failure.** |  |  |  |  |  |
| NO | 79 | [100] | 77 | [97.5] | 0.48 |
| YES | 0 | [0] | 2 | [2.53] |  |
| **Indication.for.TEE..choice.Re.evaluation.of.prior.TEE.findings.for.interval.change.** |  |  |  |  |  |
| NO | 73 | [92.4] | 76 | [96.2] | 0.49 |
| YES | 6 | [7.59] | 3 | [3.8] |  |
| **Indication.for.TEE..choice.Guidance.during.percutaneous.cardiac.interventions..ablations..valve.procedures..watchman..PFO.ASD.closures..** |  |  |  |  |  |
| NO | 66 | [83.5] | 73 | [92.4] | 0.14 |
| YES | 13 | [16.5] | 6 | [7.59] |  |
| **Indication.for.TEE..choice.Evaluation.for.aortic.dissection.transection.** |  |  |  |  |  |
| NO | 79 | [100] | 78 | [98.7] | 1 |
| YES | 0 | [0] | 1 | [1.27] |  |
| **Indication.for.TEE..choice.Evaluation.of.valvular.heart.disease.and.for.determining.planning.in.possible.interventions.** |  |  |  |  |  |
| NO | 61 | [77.2] | 60 | [75.9] | 1 |
| YES | 18 | [22.8] | 19 | [24.1] |  |
| **Indication.for.TEE..choice.Endocarditis.evaluation.and.possible.surgical.planning..could.be.for.valve.surgery.abscess.or.for.PPM.ICD.removal..** |  |  |  |  |  |
| NO | 64 | [81] | 48 | [60.8] | 0.0086 |
| YES | 15 | [19] | 31 | [39.2] |  |
| **Indication.for.TEE..choice.Evaluation.for.cardiac.source.of.embolus.** |  |  |  |  |  |
| NO | 75 | [94.9] | 73 | [92.4] | 0.74 |
| YES | 4 | [5.06] | 6 | [7.59] |  |
| **Indication.for.TEE..choice.Evaluation.for.safety.of.cardioversion.ablation.for.patients.in.afib.** |  |  |  |  |  |
| NO | 45 | [57] | 60 | [75.9] | 0.018 |
| YES | 34 | [43] | 19 | [24.1] |  |
| **Preexisting.GI.DiagNOsis.** |  |  |  |  |  |
| NO | 37 | [46.8] | 35 | [44.3] | 0.87 |
| YES | 42 | [53.2] | 44 | [55.7] |  |
| **If.inpatient..was.length.of.stay.extended.** |  |  |  |  |  |
| NO | 38 | [97.4] | 42 | [97.7] | 1 |
| YES | 1 | [2.56] | 1 | [2.33] |  |
| **X30.Day.Readmission.after.TEE.** |  |  |  |  |  |
| NO | 67 | [84.8] | 74 | [93.7] | 0.12 |
| YES | 12 | [15.2] | 5 | [6.33] |  |
| **GI.Team.Consulted.Pre.TEE.** |  |  |  |  |  |
| NO | 79 | [100] | 0 | [0] | 2.30E-35 |
| YES | 0 | [0] | 79 | [100] |  |
